# Long-Acting Luteinizing Hormone-Releasing Hormone Agonist for Ovarian Hyperstimulation Induced by Tamoxifen for Breast Cancer

**DOI:** 10.1155/2018/4931852

**Published:** 2018-01-23

**Authors:** Nobue Kojima, Yui Yamasaki, Houu Koh, Masaru Miyashita, Hiroki Morita

**Affiliations:** ^1^Department of Obstetrics and Gynecology, Rokko Island Konan Hospital, Kobe, Japan; ^2^Department of Surgery, Konan Hospital, Kobe, Japan

## Abstract

Tamoxifen treatment for breast cancer may induce ovarian cysts and supraphysiological levels of serum estrogen. We report successful management with luteinizing hormone-releasing hormone (LHRH) agonist of ovarian hyperstimulation induced by tamoxifen. A 49-year-old woman was operated on for invasive ductal carcinoma of the right breast. She received breast irradiation and adjuvant tamoxifen therapy. After 2 years, she had a cystic ovarian mass, and her serum concentration of estradiol was 1280 pg/mL. She was treated with an injection of 11.25 mg leuprolide acetate, a long-acting LHRH agonist, without abandoning tamoxifen therapy. The levels of estradiol decreased to <10 pg/mL and the cystic mass disappeared 2 months later. Three-month depot treatment with LHRH agonists can be useful for patients receiving tamoxifen for breast cancer who have ovarian cysts and supraphysiological levels of estrogen.

## 1. Introduction

Tamoxifen is a selective estrogen receptor modulator (SERM), which is widely used as hormone therapy for estrogen-receptor-positive breast cancer. It is effective for both adjuvant therapy after surgery and treatment of metastatic cancer [1, 2].

Women treated with tamoxifen have increased risks of endometrial hyperplasia and uterine cancer [3]. Moreover, tamoxifen treatment sometimes induces ovarian hyperstimulation, which causes ovarian cysts and supraphysiological levels of serum estrogen [4, 5]. Tamoxifen-induced ovarian cysts are observed in premenopausal women or in women who have amenorrhea after chemotherapy.

Some studies have shown that cotreatment with luteinizing hormone-releasing hormone (LHRH) agonists and continuation of tamoxifen resolves ovarian hyperstimulation and that LHRH agonists are given by monthly injections for 3 or 6 months [4, 5]. Three-month depot formulation of LHRH agonists may reduce the injection times if it is effective.

This report presents a rare case of a perimenopausal woman diagnosed with tamoxifen-induced ovarian hyperstimulation who had amenorrhea for 8 months, without chemotherapy for breast cancer. To the best of our knowledge, this is the first report to treat ovarian hyperstimulation with 3-month depot LHRH agonists. In addition, there are few reports about tamoxifen-induced ovarian hyperstimulation in Japanese women, and this is the first report to treat ovarian hyperstimulation without abandoning tamoxifen in Japanese women with breast cancer.

## 2. Case Presentation

**Figure 1:**
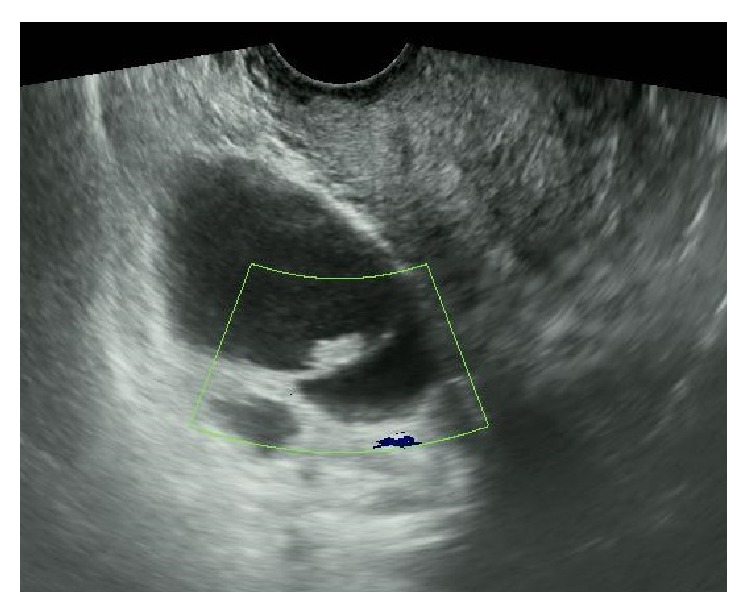
Ultrasonography demonstrated two cystic lesions in the right ovary. Color Doppler ultrasonography did not show neovascularity in the right ovary.

A 49-year-old Japanese woman, gravid 3 para 3, was referred to our hospital with a diagnosis of right ovarian cyst. She had amenorrhea for 5 months. Transvaginal ultrasonography demonstrated two cystic lesions in the right ovary (ovarian size was 49 × 33 mm). Color Doppler ultrasonography did not show neovascularity in the right ovary (Figure 1).

**Figure 2:**
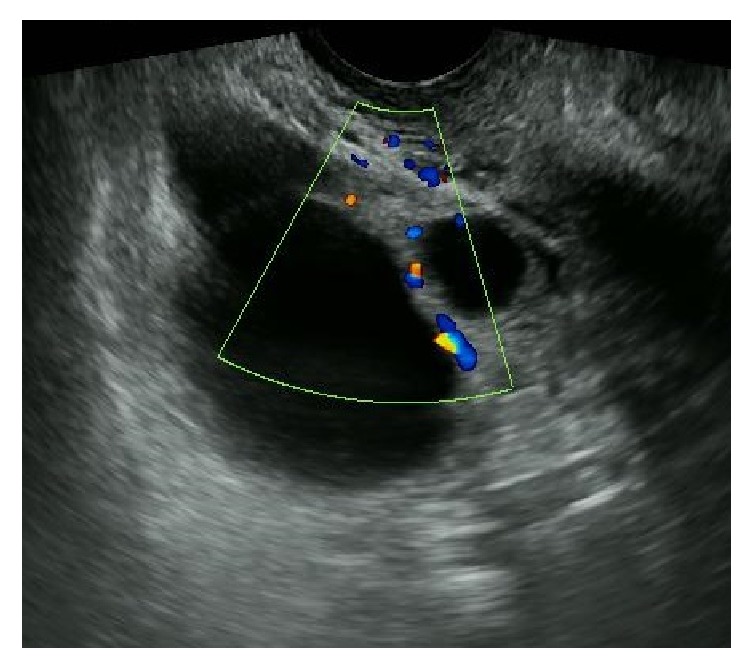
The cystic tumor of the right ovary had neovascularity demonstrated by color Doppler ultrasonography.

Three months later, there were four cystic lesions, whereas the ovarian size had not increased. At that time, color Doppler ultrasonography demonstrated neovascularity in the tumor (Figure 2). No evidence of ascites was observed. Tumor markers, cancer antigen- (CA-) 125, CA19-9, and carcinoembryonic antigen (CEA) were within the normal range. Pelvic enhanced magnetic resonance imaging (MRI) showed no evidence of malignancy.

Her medical history revealed that she was operated on for invasive ductal carcinoma of the right breast 2 years and 6 months before her first visit. Immunohistochemical test revealed that her breast cancer was estrogen receptor- (ER-) positive, progesterone receptor- (PgR-) positive, and human epidermal growth receptor 2- (HER2-) negative. After surgery, she received breast irradiation and adjuvant tamoxifen therapy (20 mg/day) without LHRH agonists. The duration of tamoxifen treatment was 2 years and 4 months. She had received gynecological check-up, and her uterus and both ovaries were normal 9 months before her first visit in the other clinic.

As the patient had received tamoxifen therapy, ovarian hyperstimulation was suspected to be the reason for ovarian enlargement. The serum concentration of estradiol and follicle-stimulating hormone (FSH) were 1280 pg/mL and 7.93 mIU/mL, respectively.

The patient had amenorrhea for 8 months, and the thickness of the endometrium was 3 mm. The right ovary (51 × 40 mm) had four follicles (the largest was 44 × 29 mm), and the left ovary (24 × 11 mm) had two follicles (both <10 mm diameter).

Informed consent for continuation of tamoxifen therapy and additional LHRH agonist therapy was received. She was treated with an injection of 11.25 mg leuprolide acetate, a long-acting LHRH agonist, without abandoning tamoxifen therapy. The levels of estradiol decreased to 51 pg/mL 4 weeks later and then to <10 pg/mL 2 months later. The ultrasonography revealed that the right ovary decreased in size, containing one follicle (27 × 20 mm) 4 weeks later, and it became normal 2 months later. These findings continued for 6 months at least.

Written informed consent was obtained from the patient for publication of this case.

## 3. Discussion

Ovarian hyperstimulation, ovarian cysts, and supraphysiological levels of serum estrogen have been reported as adverse effects of tamoxifen in 11–17% women treated with tamoxifen for breast cancer [6–8]. Tamoxifen may increase the levels of estrogen by interfering with the normal negative pituitary feedback mechanism [9] and by its direct effect with ovarian granulosa cells [10].

Tamoxifen-induced ovarian cysts are observed more frequently in premenopausal women than postmenopausal women [7]. Supraphysiological estrogen concentration is observed only in premenopausal women whose menstrual cycles in the last 3 months were regular or irregular or in women who have chemotherapy-induced menopause [8, 11]. In the present case, the patient was treated with tamoxifen without chemotherapy, and she had amenorrhea for 8 months. She seemed to be in transition to menopause; however, ovarian cysts and supraphysiological estrogen concentration were observed. It is reported that, in patients receiving tamoxifen, women with oligomenorrhea sometimes have high levels of estradiol [11] and that no relation between estradiol levels and endometrial thickness was found [12], and amenorrhea is an insufficient parameter to define menopausal status, similar to our findings.

Tamoxifen-induced ovarian hyperstimulation may cause ovarian cysts and supraphysiological levels of serum estrogen. Ovarian cysts may result from functional cysts, primary ovarian tumor including ovarian cancer, or metastasis of breast cancer. It is reported that some patients with tamoxifen-induced cysts need surgical intervention because of vascular torsion [13], cystic necrosis without vascular torsion [14], or ovarian hyperstimulation syndrome [15], or to rule out the possibility of malignant tumor. Elevated levels of serum estrogen may cause thrombotic events, endometrial hyperplasia, and uterine cancer [16]. Although the effect of supraphysiological levels of estrogen on breast cancer is unknown, increased estrogen may cause poor prognosis in women with estrogen-receptor-positive breast cancer.

When ovarian hyperstimulation is observed, the following management options can be selected: observation, surgical intervention, cessation of tamoxifen treatment, or LHRH agonist without abandoning tamoxifen treatment.

In women with breast cancer treated with tamoxifen but with no treatment for ovarian cysts, 32% of ovarian cysts increased in size and 68% decreased or completely disappeared [6]. Metastatic breast cancer and ovarian endometrioid adenocarcinoma are also reported in patients treated with tamoxifen for breast cancer [17]. If the ovarian tumor is malignant, it may progress during observation.

Surgical intervention may be needed to rule out the malignancy of ovarian tumors. Some report showed that there were malignant ovarian tumors in patients treated with tamoxifen for breast cancer [17]. On the other hand, another report showed that tamoxifen might reduce the risk of ovarian cancer in patients with breast cancer [18]. As oophorectomy reduces estrogen levels completely, it may cause long-term side-effects, including cardiovascular disease. In the present case, the possibility of ovarian cancer was considered and the results of pelvic enhanced MRI and serum tumor markers revealed that the ovarian cysts were unlikely to be malignant. In addition, after beginning the treatment of LHRH agonist, we observed ovarian cysts every 4 weeks until the cysts completely disappeared.

About 72% of tamoxifen-associated ovarian cysts disappear after cessation of tamoxifen treatment [7]. However, cessation of tamoxifen treatment might lead to poor prognosis in women with breast cancer. Ovarian cyst torsion after cessation of tamoxifen has also been reported [13].

Ovarian suppression such as LHRH agonist therapy seems to be one of the best ways to resolve ovarian hyperstimulation promptly. Three or six monthly injections of LHRH agonists can cause regression of the cysts and enable continuation of tamoxifen treatment [5]. It was reported that, within 3 weeks of the first LHRH agonist injection, serum estradiol levels fell to menopausal levels, and ovarian cysts completely disappeared within 2 months [4]. Following discontinuation of LHRH agonist cotreatment, serum estradiol levels remained at physiological levels and the ovaries remained their normal size in 64% of patients [4].

The 3-month depot formulation of 11.25 mg leuprorelin acetate produced similar pharmacodynamic effects of hormonal suppression to those achieved with monthly injections of 3.75 mg leuprorelin acetate [19]. Three-month depot leuprorelin with oral tamoxifen can suppress serum estradiol to the menopausal level within 4 weeks after injection in premenopausal women [20]. In the present case, after treatment with 3-month depot LHRH agonist, ovarian cysts and increased estrogen levels disappeared within 2 months. As LHRH agonist was needed only once, the patient could avoid receiving extra surgery.

In conclusion, ovarian cysts in women treated with tamoxifen for breast cancer may result from ovarian hyperstimulation even if she is in transition to menopause. And 3-month depot treatment with LHRH agonists can be useful in patients with ovarian cysts and supraphysiological levels of estrogen resulting from tamoxifen treatment.
